# Malignant Hypertension and the Role of Ophthalmologists: A Review Article

**DOI:** 10.7759/cureus.27140

**Published:** 2022-07-22

**Authors:** Priyadarshini Mishra, Nikita Dash, Sandip K Sahu, Vikas Kanaujia, Kumudini Sharma

**Affiliations:** 1 Ophthalmology, All India Institute of Medical Sciences, Bhubaneswar, Bhubaneswar, IND; 2 Ophthalmology, Sanjay Gandhi Post Graduate Institute of Medical Sciences, Lucknow, IND

**Keywords:** macular star, disc edema, hypertensive emergency, hypertensive retinopathy, malignant hypertension

## Abstract

Malignant hypertension (MHT) is a sudden and severe increase in systemic blood pressure (BP) associated with advanced bilateral retinopathy. It comes under a broader term, called hypertensive emergency, where an acute rise in BP results in end-organ damage. The condition usually requires hospital admission and intensive care management. Although there are lots of sophisticated machines and laboratory tests present to diagnose various organ damage, the role of ophthalmologists will still be at the top. A record of the acute rise of BP to a defining level and simple ophthalmoscopy with high clinical suspicion can save a patient’s life and preserve target organ function by timely referral. So, every ophthalmologist should be aware of this dangerous condition. In this review, we have tried to compile all the current knowledge regarding malignant hypertension that an ophthalmologist may require in day-to-day practice.

## Introduction and background

Malignant hypertension (MHT) is an acute rise in systemic blood pressure (BP) associated with advanced bilateral retinopathy. The term was first coined by Volhard and Fahr in 1914 [1] and was endorsed by Keith et al. in 1928 [2]. It comes under a broader term, called hypertensive emergency, where a severe increase in BP results in end-organ damage. Various other terminologies, like accelerated hypertension and hypertensive crisis, have been used synonymously to describe this condition with slight variations. According to the latest guidelines, a hypertensive crisis or emergency is defined by systolic BP > 180 mmHg and/or diastolic BP > 120 mmHg with target organ damage like the retina, brain, heart, large arteries, and kidneys [3-5]. The resultant systemic presentation can be a mixed picture of the following conditions such as advanced retinopathy, hypertensive encephalopathy, thrombotic microangiopathy, cerebral hemorrhage, acute stroke, acute coronary syndrome, cardiogenic pulmonary edema, aortic aneurysm/dissection, and severe pre-eclampsia and eclampsia, which usually require hospital admission and intensive care management. Although there are lots of sophisticated machines and laboratory tests at present to diagnose various organ damage, the role of ophthalmologists will still be at the top.

In this review, we have tried to compile the current knowledge on basic aspects of malignant hypertensive retinopathy as well as the latest updates in multimodal imaging and management. We will also briefly touch on other systemic manifestations of hypertensive emergency so that, in the absence of the typical bilateral fundus picture of advanced hypertensive retinopathy, the ophthalmologist should not miss the diagnosis.

## Review

Epidemiology

Malignant hypertension is uncommon in the general population. European studies found around one to two new cases per 100,000 population per year [6-9]. The incidence among African-Caribbeans is generally higher, at 7.3 per 100,000 people per year, which may be due to anti-hypertensive medication resistance and insufficient compliance with treatments [10]. Another European study provides an estimate of the prevalence of hypertensive crises in an emergency department, which accounts for 3% of the total patients but approximately one-fourth (27%) of the urgencies-emergencies [11]. Analysis of the United States data from 2000 to 2011 showed no reduction in the incidence of malignant hypertension [12]. Indian studies reflect the overall prevalence of hypertension as around 20-30% and among all hypertensives, 1-2% present with hypertensive crisis [13,14].

Several potential risk factors like female sex, the grade of obesity, the presence of hypertensive heart disease or coronary artery disease, the presence of a somatoform disorder, a higher number of prescribed antihypertensive drugs, cost, frequency of dosing, and non-adherence to the medication have been found to be associated with hypertensive crisis [15,16]. Smoking is also a risk factor in developing MHT [17,18]. Insufficient sleep, overwork, and/or mental burden of long duration were other risk factors associated with MHT [19].

Several genetic studies have been conducted to understand the mechanism of the rapid transition of a stable benign hypertensive disease to severe and devastating malignant hypertension. It shows that polymorphism in the angiotensin-converting enzyme (ACE) gene is a significant risk factor for the initiation of malignant hypertension [20].

Etiology

The majority of malignant hypertension cases are essential. Non-compliance with antihypertensive medications, differences in awareness, and less access to primary care physicians and health insurance are documented as the probable causes in Western studies [15,16,21].

In younger patients, secondary causes of malignant hypertension are more common. Several studies have shown that renal parenchymal and renovascular diseases like chronic pyelonephritis, glomerulonephritis, tubulointerstitial nephritis, polycystic kidney disease, systemic sclerosis, systemic lupus erythematosus, haemolytic uremic syndrome, renal artery stenosis, atherosclerotic diseases, polyarteritis nodosa, and fibromuscular dysplasia account for most of the secondary causes of malignant hypertension. The prevalence of these conditions varies between 11% and 67% for renal parenchymal disease and 2% and 33% for renovascular disease. The prevalence of endocrine causes such as pheochromocytoma, Conn's and Cushing’s syndrome is invariably low, accounting for less than 5% of the total number of cases [8,9,21]. Acute post-streptococcal glomerulonephritis was found to be a common cause of transient hypertension and hypertensive emergencies in Thai children [22]. In adults, neoplasm of the juxtaglomerular area and Takayasu’s arteritis involving the renal artery can cause secondary malignant hypertension [23-25].

In women, high-dose estrogen oral contraceptive pills (50-100 mcg) are associated with malignant hypertension [26]. Pre-eclampsia and eclampsia are also well-known conditions associated with MHT. Pre-eclampsia affects an estimated 4.6% of pregnancies globally [27]. Another study shows that the overall prevalence of pre-eclampsia is 2.9% and 2.3% in Sweden and China, respectively [28]. Although the incidence is on a decreasing trend due to good antenatal care, it is still not rare to be found in rural scenarios.

Pathophysiology and clinical features

An important factor in the pathophysiology of malignant hypertension is an acute, severe rise in BP. Normally, vasoconstriction occurs in response to an increase in BP. When there is a severe elevation of BP in a relatively short period of time, the autoregulatory mechanism fails, resulting in focal dilation of vessels and transmission of high BP to the endothelium. This mechanical stress damages the endothelium, causing increased vascular permeability, leakage of plasma proteins, and deposition of fibrinogen in vessel walls, thus activating mediators of intravascular coagulation and cell proliferation. It produces a vicious cycle of fibrin deposition and tissue ischemia, leading to fibrinoid necrosis of vessels.

Arterial wall hypertrophy seen in chronic hypertensive patients minimizes the transmission of pressure to the capillary circulation and relatively protects against the development of malignant hypertension unless the BP is very high.

Activation of the renin-angiotensin system, increase in circulating levels of vasopressin, endothelin, cortisol, and catecholamines; a decrease in the production of prostacyclin as a result of cigarette smoking; and immune system abnormalities are the proposed mechanisms in the pathogenesis of malignant hypertension.

In the retina, focal ischemia of the nerve fibre layer causes cotton wool spots, and the breakdown of the blood-retinal barrier results in haemorrhages, fibrinous exudates, and macular edoema. Retinal haemorrhages are usually described as striate, flame-shaped haemorrhages present in the nerve fibre layer. They are most commonly located in the peripapillary area where radial capillaries are distributed. These are long, straight, superficial capillaries from which outflow can be easily obstructed by disc and peripapillary edoema, resulting in haemorrhage.

Other causes could be pathologic changes in the capillaries like ischemic capillaropathy. Optic disc swelling occurs due to axonal hydropic swelling secondary to either ischemic infarction or increased intracranial pressure.

Hypertensive choroidopathy is more often seen in young patients with an acute rise in blood pressure. The mechanism underlying these changes is fibrinoid necrosis of the choroidal arterioles. Since choroidal arteries have a short course and supply the choriocapillaries at right angles, systemic blood pressure is transmitted directly to them. Moreover, choriocapillaris does not autoregulate as effectively as retinal circulation. This leads to non-perfusion of the overlying choriocapillaries and focal ischemic damage to the retinal pigment epithelium (RPE), manifested as Elschnig spots. Siegrist streaks are linear hyperpigmented streaks along the course of choroidal arteries, representing ischemia of choroidal lobules. Here, the choriocapillaries become attenuated in this zone, and the RPE directly overlying the choroidal arteries becomes hyperplastic. RPE pump failure due to underlying choroidopathy can result in subretinal fluid (SRF) and, in severe cases, exudative retinal detachment [29-33].

The same pathologic changes are also seen in the blood vessels of other organs. In the kidney, fibrinoid necrosis of arterioles with fine subendothelial lipid droplets and hyaline thrombi formation causes glomerular ischemia, tubular atrophy, and interstitial haemorrhage. In the brain, cerebral vasodilation, hyperperfusion, breakdown of the blood-brain barrier, plasma exudation, petechial haemorrhages, fibrinoid necrosis of cerebral arterioles, and focal edoema are the pathologic findings.

In their study, Lee et al. pointed out that pre-eclampsia-induced hypertension and malignant hypertension may not share a common pathophysiology. Pre-eclampsia patients develop choroidopathy more commonly than retinopathy and could be viewed as not having achieved a "malignant" threshold level towards the development of retinopathy [34]. This observation may be supported by the structural and functional structure of the choroidal vasculature. Anatomically, the choroidal arteries are not highly branched and run a relatively short course at a right angle to supply the choriocapillaries; thus, blood pressure is transmitted more directly to the choriocapillaries [29,35,36]. Functionally, unlike the retinal vasculature, the choroidal vasculature has few autoregulatory properties and is controlled primarily by the sympathetic nervous system. Hence, if elevated blood pressure overcomes the compensatory sympathetic response, it can damage the choroidal vasculature, whereas retinal vessels are able to tolerate much higher blood pressure while maintaining vascular tone because of autoregulatory mechanisms [37]. Other mechanisms behind this different pathophysiology are yet to be explored.

Presentation

Neurologic symptoms are often the presenting complaint in malignant hypertension. The most common are headache and dizziness, which can be seen in 60% and 28% of patients, respectively; 7% of patients can present with cerebrovascular events. In the Glasgow Blood Pressure Clinic, 17% of malignant hypertensive patients presented with the neurological symptoms of hypertensive encephalopathy. These symptoms included altered consciousness (delirium, agitation, stupor), seizures, cortical blindness, and coma.

In 35 to 60% of cases, visual impairment is the presenting symptom. It may range from bilateral mild impairment to severe loss of vision depending on the presence of only retinopathy with macular edema, subretinal fluid, or associated with macular ischemia, optic neuropathy, or severe choroidopathy leading to exudative retinal detachment. Very rarely, unilateral presentation or absent disc edoema can be seen.

Other presenting symptoms can be chest pain, gastrointestinal symptoms (nausea, vomiting, and abdominal pain), dyspnoea, peripheral edema, generalized weakness, malaise, fatigue, and epistaxis [6,8,11,32].

Ophthalmoscopy findings and their importance

**Figure 1:**
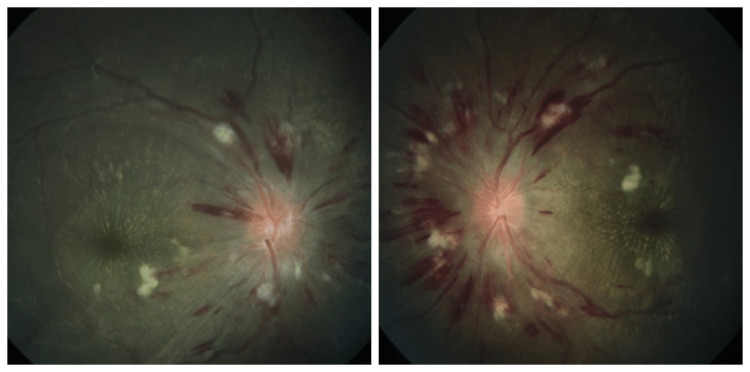
Typical fundus picture of malignant hypertensive retinopathy Fundus picture of right and left eye of a 21-year-old male patient (author's own patient) showing the typical signs of malignant hypertensive retinopathy like bilateral disc edema, peripapillary splinter haemorrhages, cotton wool spots, vascular tortuosity, venous engorgement, hard exudates at macula in form of macular star.

Hayreh et al. first described retinopathy, choroidopathy, and optic neuropathy based on an animal model of malignant renal arterial hypertension. They described an entity termed focal intraretinal periarterial transudates (FIPTs) as a retinal lesion-specific for MHT [31], but these entities could not be adequately described in humans due to difficulty in detection. Common findings seen in malignant hypertension include splinter and flame-shaped haemorrhages, cotton wool spots, disc edema, and hard exudates forming macular stars [32] (Figure 1).

Optic neuropathy can either be passive disc edoema or non-arteritic anterior ischaemic optic neuropathy (NAION), contributing to vision loss [30,38].

**Figure 2:**
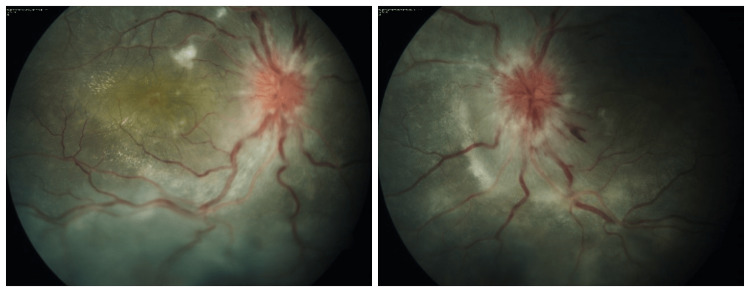
Fundus picture of a patient with malignant hypertension Fundus picture of right and left eyes of a 16-year-old male patient (author's own patient) showing signs of malignant hypertensive retinopathy like bilateral disc edoema, few cotton wool spots and splinter haemorrhages, venous congestion and tortuosity. Elschnig spots and exudative retinal detachment are present inferiorly as signs of severe choroidopathy.

Choroidopathy can be clinically identified as Elschnig spots, which are pale yellow in appearance with well-defined margins (Figure 2). Old lesions appear like pigmented spots surrounded by an atrophic pale halo. Siegrist streaks are linear hyperpigmented streaks along the course of choroidal arteries. Star-shaped hard exudates with SRF and intraretinal fluid can be seen. Severe choroidal dysfunction can lead to exudative retinal detachment [39].

The role of ophthalmoscopy in hypertension is very important. Although the sensitivity may be lower, the specificity is high, ranging from 88% to 98%, indicating hypertensive retinopathy is rarely observed in normotensive patients [40]. In severe hypertension with target organ damage, the sensitivity of a retinal examination is even higher [41,42]. A high correlation has been noted between the degree of the left ventricular mass index and the severity of hypertensive retinopathy and renal involvement [43,44]. Similarly, the risk of developing cerebral white matter lesions and clinical stroke is higher in patients with retinopathy [45].

**Figure 3:**
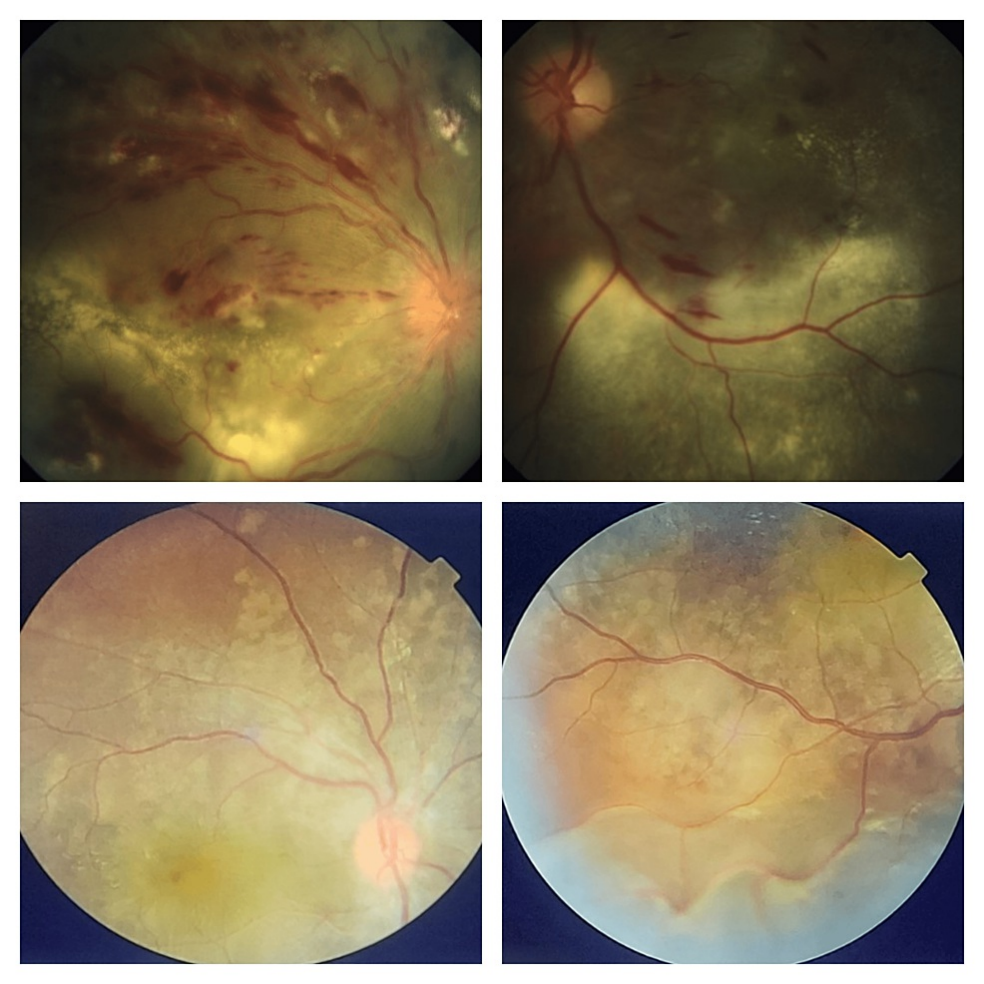
Atypical fundus pictures of patients with malignant hypertension Upper photos are fundus picture of a 41-year-old male patient (author's own patient) showing extensive subretinal fibrinous exudates inferiorly along with superficial haemorrhages and minimal disc edema. Lower photos are fundus picture of a 27 years female patient with pre-eclampsia (author's own patient) showing signs of severe choroidopathy with fibrinous deposits at macula and Elschnig’s spots and exudative retinal detachment but no signs of retinopathy like haemorrhage, cotton wool spots or hard exudates.

In malignant hypertension, the fundus findings are usually bilateral, but sometimes diagnostic confusion may arise due to unilateral or asymmetrical, or atypical pictures like the absence of disc edoema, incomplete or absent macular star, only exudative retinal detachment in the absence of haemorrhage or cotton wool spots, the presence of extensive fibrinous exudates, etc. (Figure 3).

In those cases, careful history, examination, and systemic investigations for other end-organ damage like electrocardiography (ECG), haemoglobin, platelet count, fibrinogen, creatinine, etc., can be added to fundoscopy. More specific tests such as troponin, creatinine kinase myocardial band (CK-MB), echocardiography, computerized tomography (CT), or magnetic resonance imaging (MRI) scan of the brain are done when indicated. Cremer et al. [46] have suggested hypertension-mediated organ damage (MOD) as a new terminology to describe other end organ damage.

Classification and grading

The Keith-Wagner-Barker [47] and Wong-Mitchell [48] classifications are accepted worldwide for grading hypertensive fundus changes. However, there are no such classifications for malignant hypertensive changes. Patients with grade-III or grade-IV hypertensive changes, with other evidence of end-organ damage or severely raised systolic and/or diastolic BP, have often been clubbed as malignant hypertensive retinopathy. Ahn et al. have proposed an optical coherence tomography (OCT) based classification depending on the presence or absence of subretinal fluid [49].

Newer imaging modalities and changing role of ophthalmologist

 

Routine fundoscopy usually gives an adequate clue towards the diagnosis of malignant hypertension. Fundus imaging can be done for documentation purposes. The main role of newer diagnostic tools is to prognosticate visual as well as systemic outcomes at presentation and at follow-up.

Traditionally, fundus fluorescein angiography (FFA) is used to detect macular ischemia. Increased foveal avascular zone (FAZ), delayed choriocapillaris filling, severely delayed retinal arterial filling, and capillary bed changes, i.e., dilation, closure, and leakage, are appreciated, particularly in the vicinity of soft exudates. Acute Elschnig spots have better visibility with fluorescein angiography [50]. Indocyanine green angiography (ICGA) demonstrates hypocyanescence of ischemic areas of the choroid. The larger area of choroidal filling defect is appreciated in ICGA than in FFA [51,52].

Optical coherence tomography and optical coherence tomography angiography

**Figure 4:**
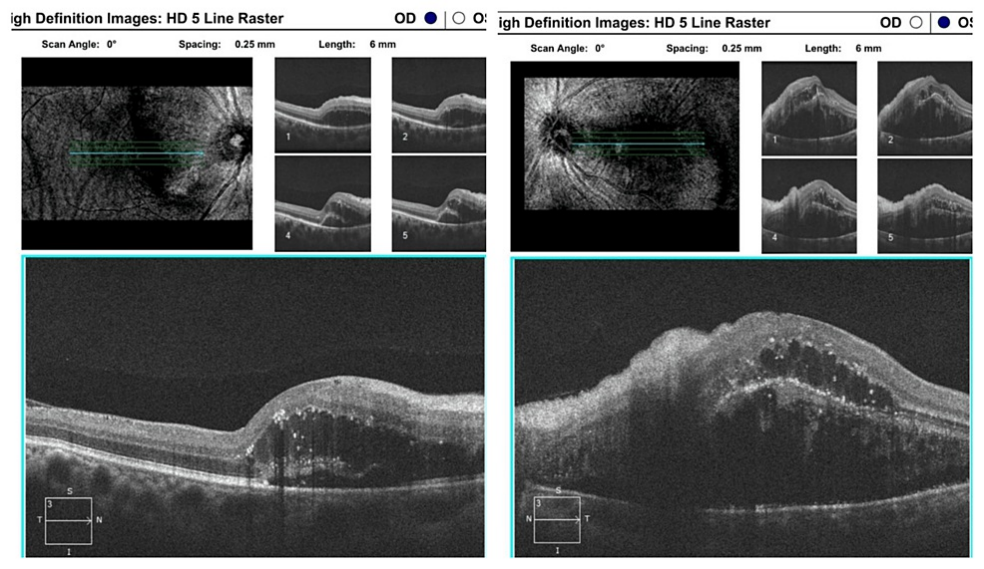
Optical coherence tomography scan of macula in a patient with malignant hypertension OCT macular scan of both eyes of a patient (author's own patient) with malignant hypertension showing bilateral nerve fibre layer thickening and wrinkling, subretinal fluid, cystoid macular edema, hyperreflective dots, disorganization of retinal outer layer

Morphological and structural abnormalities of the retinal layers due to severe hypertension are better visualized by OCT. Newer OCT machines with spectral-domain (SD) and swept-source (SS) technologies provide better image resolution that helps in the detailed analysis of the retina and choroid [53]. Features of macular edoema, irregular reflection or thickening of the retinal nerve fibre layer (RNFL), SRF, inner retinal fluid, and hyperreflective dots within the retina are observed in severe hypertension. Irregular reflections from the retinal nerve fibre layer may be caused by ischaemic damage to the nerve fibre layer and flame-shaped haemorrhages. Hyperreflective dots within the retina are often found in the outer nuclear layer, but their location can vary from subretinal space to the ganglion cell layer. Intraretinal fluid in the outer nuclear layer is commonly seen [53-55] (Figure 4).

Although subfoveal choroidal thickness (SFCT) decreases with age in patients with chronic hypertension, it is thicker in patients with hypertensive retinopathy [56,57]. An increase in SFCT in the acute phase of hypertensive choroidopathy has been attributed to hyperperfusion of the choroidal circulation [58].

Optical coherence tomography angiography (OCTA) has allowed for the non-invasive assessment of retinal and choroidal microvasculature. OCTA has been used to evaluate the superficial and deep capillary density and en face images of the macula. It can also assess the optic nerve head and radial peripapillary capillary density. The vascular flow is evaluated in both the macular and disc regions. Based on OCTA findings, the hypertensive crisis is associated with a significant reduction in retinal and choroidal capillary perfusion. These alterations are independent of retinopathy and related to end-organ damage. Focal ischemia in the choriocapillaris and reperfusion after control of BP have also been noted in pregnancy-induced hypertensive choroidopathy [58,59]. At the same time, some studies have also demonstrated that choroidal blood flow velocity and thickness concurrently increase in the acute phase of hypertensive chorioretinopathy, suggesting the role of choroidal hyperperfusion in the pathogenesis of hypertensive chorioretinopathy [60]. The mechanism underlying this difference in choroidal perfusion is yet to be understood.

Fundus autofluorescence (FAF) is a non-invasive modality to detect alterations in RPE [61]. It can demonstrate changes secondary to hypertensive choroidopathy. Ramezani et al. first described FAF changes in patients with chronic essential hypertension. They suggested that a ring of hyperautofluorescence in the central macula forming a doughnut-shaped feature might be a FAF sign in cases suffering from chronic essential hypertension lasting for more than five years [62]. Elschnig spots are described as hypoautofluorescent lesions, suggesting RPE necrosis and loss of lipofuscin [62]. FAF imaging of Siegrist streaks present with linear streaks of granular AF within the arcade. They heal with depigmentation of lesions, causing hypoautofluorescence on follow-up [63].

Prognosis

Typical retinal changes rapidly disappear within two to three months with prompt initiation of antihypertensive treatment, but not all malignant retinopathies are entirely reversible. Although cotton-wool spots resolve within one month of BP control, they are clinically significant as they represent permanent nerve fibre layer defects. Intraretinal hyperreflective dots have been known to persist for more than six months in some patients. Poor visual recovery on follow-up, despite resolution of SRF, has been attributed to photoreceptor defects better appreciated in OCT as focal loss of interdigitation zone (IZ) and ellipsoid zone (EZ) [55,56].

An association has been reported between the development of irreversible ocular complications and very high BP at the time of diagnosis, with a prominent visual disturbance at presentation and a prolonged duration of the symptoms [64]. Optic nerve infarction and choroidal neovascularization also have an effect on visual outcomes [65].

Treatment

Malignant hypertension is considered a hypertensive emergency, mandating an immediate and controlled reduction of blood pressure to a safe level. A reduction in blood pressure by one-third of the total reduction planned is reasonable during the first six hours, a further third over the next 12-36 hours, and the final third slowly over the following 48-96 hours. These patients are best treated in an intensive care unit with titratable, short-lived, and rapid-acting intravenous (IV) hypotensive agents like clevidipine, labetalol, esmolol, fenoldopam, nicardipine, and sodium nitroprusside. Oral antihypertensive treatment is progressively introduced on the clinician’s advice [66-68]. Rapid lowering of blood pressure to more than 50% makes the patient with malignant hypertension prone to cerebral hypoperfusion, ischemic stroke, and death [69,70].

Ocular changes usually resolve with systemic anti-hypertensive medications. Intravitreal bevacizumab (1.25 mg/0.05 ml) injection showed rapid resolution of the macular and optic disc edoema in the exudative stage of hypertensive retinopathy [71,72]. Resolution of retinal changes in the fellow eye has also been reported [73].

Discussion

This incidence and prevalence appear to be higher in clinical practise than those documented as malignant hypertension or hypertensive emergencies. In the Indian scenario, this may be particularly relevant to secondary malignant hypertension where the diagnosis written on the discharge paper is usually the causative disease. So unless we thoroughly search the patient’s documents, this diagnosis may be missed. Secondly, when a patient comes with complications of malignant hypertension requiring admission and intensive care management, an immediate bedside fundus examination should be done to know the true incidence of malignant hypertensive retinopathy as the retinal changes are reversible in the majority of cases with proper treatment. Large multicentric studies should be designed to know the actual incidence of this so-called uncommon disease.

There are still a lot more questions that are yet to be answered. Is there any difference in optic nerve head structure at an ultrastructural or genetic level that pre-disposes to ischemic optic neuropathy in some and only passive edoema in others? What is the exact pattern of choroidal perfusion during a hypertensive crisis? Does the pathophysiology of pre-eclampsia/eclampsia differ from secondary malignant hypertension due to other causes? Further research in this line can help us better understand the prognosis of this entity.

## Conclusions

Malignant hypertension needs emergency intervention. Although the diagnosis and management of hypertensive retinopathy, including malignant hypertension, has not changed much in these years, the chance of misdiagnosis of malignant hypertensive retinopathy is still high due to its varied presentation. In day-to-day patient care, this entity should not be forgotten as a differential diagnosis, especially when first encountered by an ophthalmologist without a previous history of hypertension or any systemic disorder. A record of the acute rise of BP to a defining level and simple ophthalmoscopy with high clinical suspicion can save a patient’s life and preserve target organ function by timely referral.

In the present situation, the role of the ophthalmologist has gone far beyond diagnostic responsibility. With the help of multimodal imaging, we can now predict visual prognosis as well as monitor the effect of systemic treatment. The correlation between ocular findings and other target organ damage is already established. Hopefully, we can predict the systemic prognosis as well in the future. This article will definitely inspire young researchers to explore more in this field.
